# *PDE1A* polymorphism contributes to the susceptibility of nephrolithiasis

**DOI:** 10.1186/s12864-017-4247-8

**Published:** 2017-12-20

**Authors:** Zhenxing Yang, Tao Zhou, Bishao Sun, Qingqing Wang, Xingyou Dong, Xiaoyan Hu, Jiangfan Zhong, Bo Song, Longkun Li

**Affiliations:** 1 Department of Urology, Second Affiliated Hospital, Third Military Medical University, Chongqing, People’s Republic of China; 2 Department of Urology, Southwest Hospital, Third Military Medical University, Chongqing, People’s Republic of China; 3 Ostrow School of Dentistry and Department of Pediatrics, School of Medicine, University of Southern California, Los Angeles, CA USA

**Keywords:** PDE1A, Nephrolithiasis, Polymorphism

## Abstract

**Background:**

Previous studies have confirmed a family risk of nephrolithiasis (NL), but only 15% of all cases are associated with an identified monogenic factor. In clinical practice, our group encountered a patient with NL combined with cystic kidney disease that had 3 affected family members. No known mutations association with NL was detected in this family, and thus further investigation of the molecular cause of NL was deemed to be necessary.

**Results:**

Quality analysis from the sequencing stage showed a more than 80-fold average depth and 95% coverage for each sample, and six mutations within six genes were chosen as candidate variants for further validation. Genotyping of rs182089527in the phosphodiesterase 1A (*PDE1A*) gene in the validation cohort indicated that the alternative allele was present in 15 patients with heterozygosity and in 1 patient with homozygosity, and exhibited significant enrichment in NL patients (Fisher’s exact test, adjusted *p* = 0.0042) and kidney cystic patients (Fisher’s exact test, adjusted *p* = 0.067) compared to controls. In addition, function analysis displayed a significant decrease in the protein and mRNA expression levels resulting from the rs182089527 mutant sequence compared with the wild-type sequence. Moreover, patients with this mutation displayed a high level of creatinine and urea in urinalysis.

**Conclusions:**

Our study provides genetic evidence that the rs182089527 mutation in *PDE1A* is involved in the development of NL and kidney cysts, which should help to improve personalized medicine for diagnosis and treatment.

**Electronic supplementary material:**

The online version of this article (dio: 10.1186/s12864-017-4247-8) contains supplementary material, which is available to authorized users.

## Background

Nephrolithiasis (NL), also known as kidney stones, is a common disease associated with major morbidity because of colicky pain, the necessity of surgical procedures, and even acute renal failure [1]. The estimated life time prevalence of NL is about 1–15% worldwide, which varies according to age, gender, race, and geographic location. In China, the overall prevalence rate was estimated to be 4.0% (4.8% in men and 3.0% in women), and the prevalence was different between northern (4.1%) and southern regions (4.0%) [2]. Both genetic and environmental factors contribute to the risk of NL. In a genetic epidemiology follow-up investigation of a cohort of 37,999 male participants, the relative risk of the incidence of stone formation in men with a positive family history was 2.75 (95% confidence interval = 2.19–3.12) [3].A twin study in Vietnam indicated that the proband concordance rate in monozygotic twins (32.4%) was significantly greater than the rate in dizygotic twins (17.3%), and the estimated heritability of the risk for NL was 56%.

The genetic cause of NL is complicated, ranging from the rare monogenic to the more common polygenic forms. To date, at least 30 genes have been reported to be associated with Mendelian forms of NL or nephrocalcinosis by autosomal-recessive, autosomal-dominant, or X-linked transmission [4, 5].Several renal calcium stone-related disorders are known to be monogenic, such as Dent disease (Online Mendelian Inheritance in Man [OMIM 300009]), Bartter’s syndrome, idiopathic hypercalciuria, primary distal renal tubular acidosis, hypophosphatemic rickets with hypercalciuria, familial hypomagnesemia with hypercalciuria and nephrocalcinosis, primary hyperoxaluria, cystinuria, renal hypouricemia, and hypocalciurichypercalcemia [6], but only about 15% of 272 genetically unrelated individuals were shown to have single-gene disease which have mentioned above [7]. With the development of microarray technology, genome-wide association studies have also been employed to investigate the susceptibility genes of NL. One such study conducted by Gudmar et al. [8] identified common, synonymous variants in the *CLDN14* gene that were associated with kidney stones, and homozygous carriers of the single nucleotide polymorphism (SNP) rs219780[C] in *CLDN14* were estimated to have a 1.64-times greater risk of developing the disease compared to non-carriers. Another study conducted in Japan found three novel loci for NL and revealed the association of SNP rs11746443 (upstream of the *SLC34A1* gene) with the reduction of estimated glomerular filtration rate [9]. Although dozens of susceptibility genes have been reported, in-depth analysis of the monogenic causes of NL is still necessary.

**Fig. 1:**
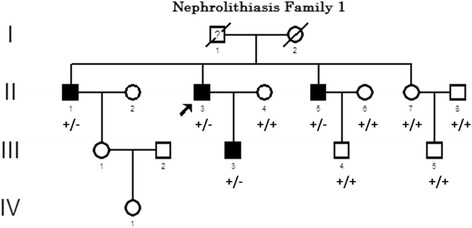
A pedigree of nephrolithiasis family with affected patients (blackened symbols) and *PDE1A* gene mutation which located in chr2:183,106,640. Symbols with question mark denote family members who are probably affected. Circles represent female family members, squares represent male family members, and crosses represent dead family members. Wilde-type and mutant-type are marked by a plus sign (+) and a minus sign (−). Arrows indicate probands

With the advent of next-generation sequencing, the mutation analysis of Mendelian genes has not only become technically available but is also cost-effective. In clinical practice, our group encountered a family affected by NL (Fig. 1), with four family members affected. Proband DNA samples were collected and sequenced initially, but no known association mutation of NL was detected.

In addition, renal cysts are extremely common and are present in more than one third of NL patients older than 50 years. More importantly, NL shows a common complication of kidney cyst development. Therefore, to further explore the potential causal genes, we employed whole-exome sequencing technology of blood samples from 10 family members (4 affected and 6 unaffected) and 2307 validation samples (including 993 NL patients and 1314 controls, 328 kidney cystic patients and 622 controls) to investigate the causal mutations, and postulated that NL may be a monogenic disease. Following functional validation was conducted using RT-PCR and western blot.

## Methods

### Sample collection

#### Proband and nephrolithiasis family description

A elder man presented with post-polio syndrome of right foot claudication,20 years history of renal calculi, repeated bilateral back pain, urgent micturition, hematuria and dysuria. The patient suffered from hypertension almost 10 years, admitted oral antihypertensive drugs, and also complained with intermittent voiding stones in urine 3 times, but was not treated. He had noticed with aggravation of back pain and voiding stones in urine again 1 month ago, which make them admitted to our Urology Department. Physical examination revealed a temperature of 36.3 °C, pulse of 102, blood pressure (BP) of 109/88 mmHg, left flank tenderness and a palpable left kidney but was otherwise normal. Laboratory testing indicated that creatinine concentration was 89.9 umol/L, the serum calcium was 2.26 mmol/L, uric acid was357 umol/L, serum phosphorus was 1.06 mmol/L, and serum magnesium was 0.78 mmol/L. The serum electrolytes were within normal limits. Urinalysis showed specific gravity of 1.02, PH 6.5, no protein, and 6 to 10 white blood cells and red blood cells (RBCs) per high-power field without casts or crystals. Abdominal ultrasound showed multiple cystic dark areas with the maximum diameter of 2.2 cm, clear boundaries and regular morphology. Ultrasonography and computed tomography (CT) of left kidney demonstrated hydronephrosis, no marked parenchymal atrophy, and multiple stones in therenal pelvis. There was a cystic dark area, diameter of 1.1 cm, in right kidney with regular morphology and clear boundary (Additional file 1: Figure S1).The patient has endured family history of nephrolithiasis (Fig. 1).

#### Cohort samples

Nine hundred ninety three cases of nephrolithiasis (NL) and 1314 controls were obtained from the Second Affiliated Hospital of Third Military Medical University during the period of 2013–2016. Diagnostic evaluations were performed separately in person using standardized criteria for diagnosing nephrolithiasis. Two independent raters collected family history from the proband, each participating parent, and a nursing sister recorded pedigree information during the clinical interview. In addition, 328 cases patients with simple cyst and 622 matched (1:2) controls were recruited for PDE1A genotype analysis. All of the cases were diagnosed by spiral computed tomography (CT), ultrasonography or X ray. Control inclusion criteria: ①Screen for a lifetime absence of NL or cyst using CT, ultrasonography or X ray. ② Excluded significant complication disease of NL or cyst. This study was conducted with the approval of the ethics committee of Third Military Medical University. whether the participants had the capacity to consent was evaluated by the following criteria: Firstly, patients have the ability to understand and reasoning; Secondly, patients have the ability to make rational decisions. All participants gave written informed consent; parental consent for children who were younger than 18 years old was obtained.

### DNA and RNA extraction and quality controls

Genomic DNA was extracted from peripheral blood cells using TIANamp Blood DNA Kit (TIANGEN BIOTECH, BEIJING) following as the manufacturer’s instructions. Total RNA was extracted from whole blood with MagMAX™ for Stabilized Blood Tubes RNA Isolation Kit following the manufacturer’s instructions. Quality control of DNA or RNA were conducted by Agarose gel electrophoresis and λDNA-Hind III digest band (Additional file 1: Figure S2).

### Captured library construction, Clustering & Sequencing

Each sequenced sample was prepared according to the Illumina protocols. Briefly, onemicrogram of genomic DNA was fragmented by nebulization, the fragmented DNA isrepaired, an ‘A’ is ligated to the 3′ end, Illumina adapters are then ligated to the fragments, and the sample was size selected aiming for a 350–400 base pair product. The size selectedproduct was PCR amplified, and the final product was validated using the Agilent Bioanalyzer. Two steps of hybridization and wash were needed for construction. PCR was used in order to amplify the enriched DNA library for sequencing. PCR was performed with the same PCR primer cocktail used in TruSeq DNA Sample Preparation. Axeq Technologies performs procedures for quality control analysis on the sample library and quantification of the DNA library templates. Illumina utilizes a unique “bridged” amplification reaction that occurs on the surface of the flow cell. A flow cell containing millions of unique clusters was loaded into the HiSeq 2004 for automated cycles of extension and imaging. Following cluster generation, 151 nt paired-end sequencing was performed using the standard Illumina protocols.

### Quality control for sequencing results and variants calling

Quality control of raw data was conducted by FastQC software (http://www.bioinformatics.babraham.ac.uk/projects/fastqc/). Reads were mapped to a custom hg19 build using Burrows-Wheeler Alignment tool (BWA).The duplicate reads were flagged using Picard-tools (http://broadinstitute.github.io/picard/). GATK IndelRealigner module was used to realign reads around insertion/deletion (Indel) sites. Individual sequence data (in BAM format) was preprocessed as whole NGS community suggests which mainly include local Indel realignment, PCR duplicates removal and base quality recalibration. Read qualities were recalibrated using GATK Table Recalibration. GATK unified Genotyper module was then used to call variants (both SNVs and Indels) from multiple samples simultaneously, which create a single Variant Call Format (VCF) file. Raw read data were visualized using the Integrative Genome Viewer (IGV).Individual level quality control was conducted on raw and clean variant to make sure avoiding false positive variants. A suite of per-individual metrics, which included the total number of alternate alleles, dbSNP coverage (build137), and Transition/Transversion (Ti/Tv) ratio, and variant quality recalibration (VQSR) were calculated. From available exome data, we extracted common variants and estimated per-individual heterozygosity (~inbreeding), pairwise relatedness, and sex-check using PLINK (Additional file 2: Table S1). Variants quality control was conducted by software KGGSeq (http://statgenpro.psychiatry.hku.hk/limx/kggseq/ doc/UserManual.html), which were carefully designed to filter and prioritize gene variants in exome sequencing of rare Mendelian and common complex disorder.

Candidate mutation from the next generation sequencing were tested using standard Sanger sequencing on an ABI 3730xl DNA Analyzer to validate the reality, by designing custom primers (Sigma) based on ~200 bp of genomic sequence flanking each variant (Additional file 1 **:** Figure S3).

### Variance quality control and candidate gene filtering

Variants were kept if ① the minimum overall sequencing quality scores ≥ 50 (−-seq-qual 50) and the minimum overall mapping quality score ≥ 20 (−-seq-mq 20);②The minimal genotyping quality per genotype ≥ 30 (−-gty-qual 30) and the minimal read depth per genotype ≥ 30 (−-gty-qual 30); ③The fraction of the reads carrying alternative allele ≤ 5% at a reference-allele homozygous genotype (−-gty-af-ref. 0.05), the fraction of the reads carrying alternative allele ≥ 25% at a heterozygous genotype (−-gty-af-het 0.25), and the fraction of the reads carrying alternative allele ≥ 75% at an alternative-allele homozygous genotype (−-gty-af-alt 0.75); ④ Minimal observed number of non-missing genotypes in all samples as 50 (−-min-obs 50).

Candidate variants were kept if ① mutation present in patients with nephrolithiasis, but not in family members. ② Nonsynonymous; All mutations were annotation by Annovar, we ignored synonymous variants because nucleotide substitution of these kind variants does not change amino acid. ③ Predict damaging; All of nonsynonymous variants that met any of the following criteria were considered potentially damaging: frameshift, nonsense, stoploss, stopgain, splicing and missense mutation with Polyphen score≧0.90 and/or SIFT p≦0.05 and/or Grantham score≧100 and/or phyloP score≧2.0. ④ SNP or In/Del within a gene, which have been proved with the cause of nephrolithiasis or related pathway in previous study.

### Genotyping

Six SNPs were chosen for further validation which based on the prediction scores, minor allele frequency (MAF) and function annotation (Additional file 2 **:** Table S2). The SNP genotyping was performed using an improved multiplex ligation detection reaction (iMLDR) technique or TaqMan, which was newly developed by Genesky Biotechnologies, Inc. (Shanghai, China). We designed primers and probes for TaqMan genotyping assays for SNP rs182089527. Each genomic DNA sample (20 ng) was amplified with TaqMan universal PCR master mix reagent (Applied Biosystems, Foster City, CA) combined with the specific TaqMan SNP genotyping assay mixture, corresponding to the SNP to be genotyped. The iMLDR technique was applied for genotyping remaining SNPs which follow the instruction.

The sequencing of PDE1A designed primers as following:

Upper primer:TGACCTCTCACATATGCTGCTGT.

Lower primer:TTGGTGAGCTCTCTTGGATCA.

### Functional studies

#### Construction of mutation expression plasmid

Blood samples were collected from all family members. RNA were extracted and was converted into complementary DNA (cDNA) using a Reverse Transcription System Kit (Invitrogen, Carlsbad, CA, USA). Wild-type and mutant-type of p.M1R in PDE1A9 were harvested from 6 unaffected members and 4 affected patients.

cDNAs were PCR-amplified with forward primerand reverse primer (5- TACCGGACTCAGATCTCGAGCGCCACCAGGGGCAAAAAGATAAACAAAC-3 and 3-GATCCCGGGCCCGCGGTACCGTCTGATGAATAAACTCACACTTCTG-5). Human wild-type and mutant PDE1A9 inserted into the GV320 vector (SHANGHAI GENECHEM CO., LTD.). The viruses were collected on Day 3 after the transfectionand were concentrated by ultracentrifugation.Transport plasmid with human wild-type and mutant PDE1A9 constructs were transiently or stably transfected intoTubular epithelial cells (ATCC, Manassas VA, USA; CCL-93). All constructs were verifiedby sequencing.

#### Cell culture

All the tubular epithelialcell lines were provided by prof. Jing Zhang and her student Yi Gong (Department of Nephrology, The Third Millitary University) and cultured in DMEM supplemented with 10% fetal bovineserum, streptomycin (100 mg/ml) and penicillin (100 U/ml). Cells wereserum deprived for 6 h before treatment with transport plasmid andthen cultured in DMEM containing 0.5% fetal bovine serum (conditionedmedium).

#### Western blot

Protein was extracted from cultured tubular epithelial cell lines after washed with PBS and lysed in RIPA buffer (Beyotime, Shanghai, China). Protein concentration was measured using the RCDC method (BIO-RAD, USA). Total protein extracts of 50 μg were separated using 8–10% SDS-PAGE gels (Beyotime, Shanghai, China) and transferred to polyvinylidene fluoride membranes (Millipore, Billerica, USA). After blocked with 5% BSA at room temperature for 2 h and the membraneswere incubated with primary antibodies against PDE1A9at 4 °C overnightfollowed by the secondary antibodies. Proteins were visualized using ECL (Millipore, Bradford, MA, USA) and detected using Image Quant LAS-4000 (Fujifilm, Tokyo, Japan) BioImaging System.

#### RT-PCR

Cultured tubular epithelial cells were lysed on the ice for 20 min. cDNAs were synthesized by Sensiscript RT Kit (Qiagen, Hilden, Germany) according to the manufacturer’s instructions. The primers was the same as above description. The thermocycling program consisted of 94 °C for 1 min, 60 °C for 30 s and 72 °C for 1 min (40 cycles).The PCR products were visualized using 2% agarose gel electrophoresis followed by GoldView (SBS Genetech Co., Beijing, China) staining.

### Statistical analysis

Clinical characteristics are presented as means ± SD. The chi-squared test or Fisher’s exact test with Bonferroni correction was used for the analysis of contingency tables depending on the sample size. Monte Carlo simulation was employed to calculate the difference between the NL patients with the rs182089527 mutation and healthy control group. All statistical analyses were conducted using R software (http://www.R-project.org/).

## Results

### Sequencing

We enriched exonic sequences from the 10 family members using the Illumina TruSeq Exome Enrichment system for targeted exome capture, and Illumina paired-end sequencing was performed. On average, 61.09 Gb of mappable sequence data per individual were obtained after exome enrichment, targeting ~74.86 Mb from the exons and their flanking regions. The paired-end reads were aligned to the reference genome (hg19 build) using the Burrows-Wheeler Aligner (BWA) [10]; 98.5% of the reads were correctly aligned to the reference genome. The median read depth was 82×, which was higher than the estimated depth (33×) for highly accurate downstream heterozygous variant calling. In addition, 94.37% of the captured target exomes were covered by high-quality genotype calls at least 20 times to ensure properly detection sensitivity [11] (Additional file 2: Table S1). Overall, we covered about 2.39% of the genome, a fraction corresponding to the National Center for Biotechnology Information Consensus Coding Sequences database. After prioritization of the candidate variants list, six SNPs within six genes (*PDE1A*, *CERKL*, *MAN2B2*, *CYP1A2*, *NAGLU* and *NFATC1*) were chosen for further validation (Additional file 2: Table S2).

### Clinical characterization and genotyping

The gender and age distributions did not differ significantly between either 993 NL cases and 1314 controls, or 328 kidney cystic patients and 622 controls. There was a significant difference in the frequency of SNP rs182089527 in the phosphodiesterase 1A gene (*PDE1A*) between the NL patients and controls (Fisher’s exact test, *p* = 0.0003, Bonferroni correction *p* = 0.0042) (Table 1). Data obtained from the 1000Genome database showed that the minor allele of rs182089527 is C, with a frequency of 0.26%, indicating that only 13 of 2500 individuals carry this mutation [12]. In the current investigation, 19 of the 2307 total samples showed the rs182089526 variant allele C with a minor allele frequency (MAF) of 0.43%, which is slightly higher than that of the global population in the 1000 Genomes Project database, with significantly greater enrichment in the case group (chi-squared test, *p* = 0.0012, odds ratio [OR] =3.31). More importantly, the rs182089527 distribution also showed a trend of difference between kidney cyst patients and healthy controls for both the allele (Fisher’s exact test, *p* = 0.0048, adjusted *p* = 0.067) and genotype (Fisher’s exact test, *p* = 0.0049, adjusted *p* = 0.068) comparisons.

**Table 1:** SNP genotyping of rs182089527 within PDE1A in cohorts

rs182089527	Nephrolithiasis (*N* = 993)	Normal (*N* = 1314)	*P* value	OR	95%CI	Adjust P^a^
AA/AC/CC	977/15/1	1310/3/0	0.0003			0.0042
A/C	1969/17	2623/3	0.00015	7.55	[2.21,25.8]	0.0021
	Cystic (*N* = 328)	Normal (*N* = 622)				
AA/AC/CC	323/5/0	622/0/0	0.0048			0.0672
A/C	651/5	1244/0	0.0049	∞	[1.74, ∞]	0.0686

*OR* Odds Ratio

^**a**^Bonferroni correction for original P value

### Functional and expression analyses in tubular epithelial cells

To determine whether the rs182089527 mutation results in a change in the expression level of *PDE1A* isoform 5, we measured the protein and mRNA expression levels between tubular epithelial cells injected with the wild type (WT) and rs182089527 mutant sequence. Western blot and reverse transcription-polymerase chain reaction (RT-PCR) results indicated a significant decrease (*P* < 0.001) in the protein and mRNA expression levels of cells transfected with the rs182089527 mutant sequence compared with those carrying the WT type sequence (Additional file 1: Figure S4). The data were derived from a minimum of 40 tubular epithelial cells with 3 biological replications per experiment.

### Genotype-phenotype correlations

**Fig. 2:**
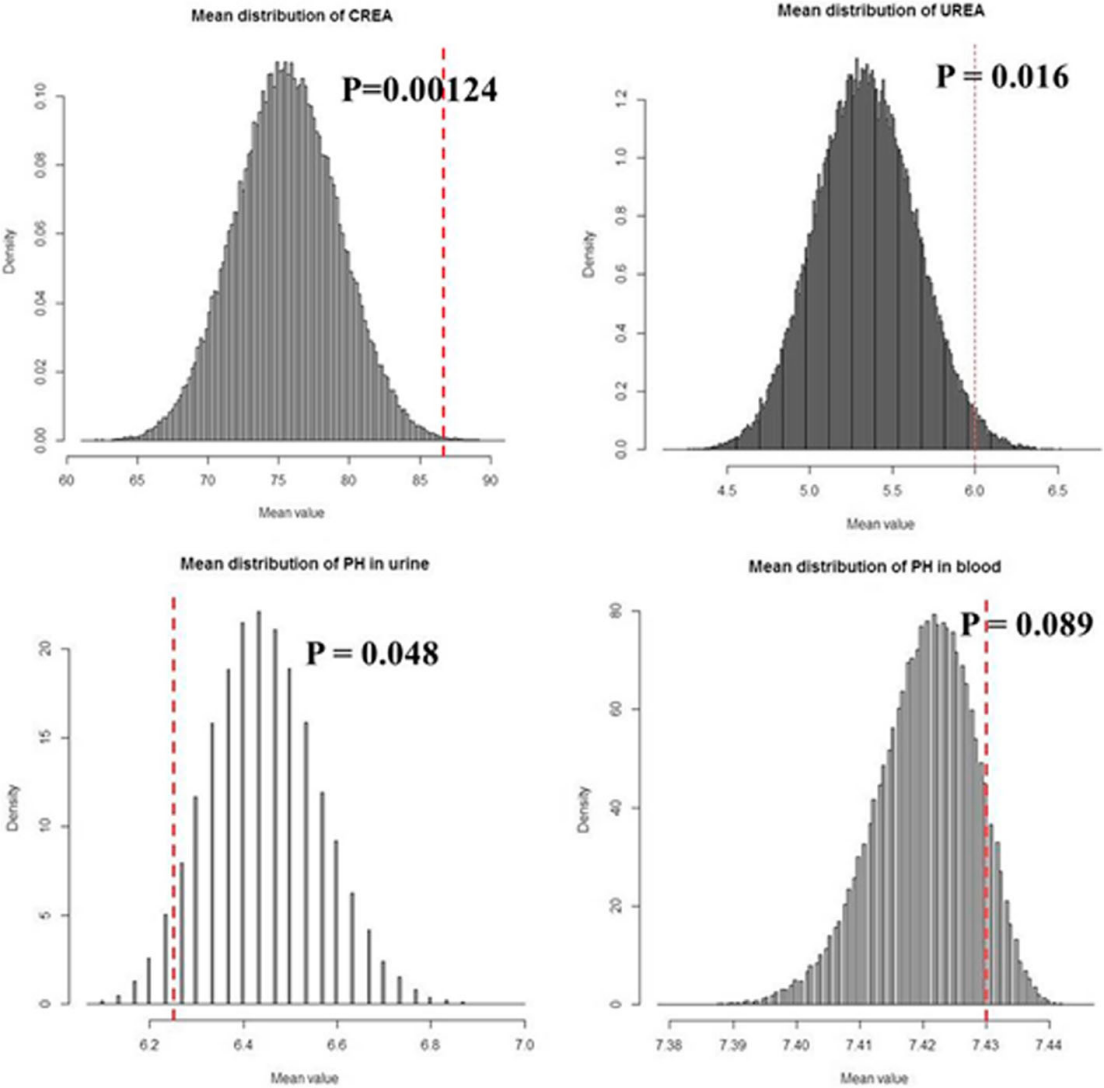
Randomly choose 19 values of clinical experimental test in patients from 894 values of clinical experimental test in normal controls got an empirical distribution of average values for different clinical experiment lab test. The red line denoted the average values from 19 samples with rs182089527 mutation. CREA, creatinine

A total of 19 patients with NL displayed the C allele mutation in the *PDE1A* gene. Only two of 19 patients were subjected to stone component analysis, revealing calcium oxalate stones. Urinalysis indicated that all of 19 patients had high level of creatinine (Monte Carlo simulation, *p* = 0.0012) and urea (Monte Carlo simulation, *p* = 0.016). The pH level differed slightly between the cases and controls in the urine (lower in cases, *p* = 0.048) and blood (slightly higher in cases, *p* = 0.089) (Fig. 2). No other laboratory findings showed difference in these subgroups (Additional file 1: Figure S5).

## Discussion

Initial analysis of a pedigree family affected by NL revealed a loss-of-function mutation in the *PDE1A* gene that contributed to the susceptibility of NL. This mutation was also detected in a second cohort of patients suffering from NL, at a frequency of 1.61%. Transfection of a plasmid with cDNA expressing thers182089527 mutation in a tubular epithelial cell line revealed the complete loss of function of *PDE1A* isoform 5 (also known as *PDE1A9*).

To our knowledge, this is the first study to investigate the genetic cause of cystic kidney disease. Previous studies suggested an association of genetic factors with autosomal-dominant transmission, but were lacking in detailed data. In the current study, we detected the rs182089527 mutation in *PDE1A* in 5 patients suffering from cystic kidney disease. The *PDE1A* gene, located within chromosome 2q32.1, includes 23 exons and encodes 9 isoforms of *PDE1A* through alternative splicing [13]. *PDE1A* belongs to the family of cyclic nucleotide phosphodiesterases (PDEs) that play an important role in signal transduction by regulating the intracellular cyclic nucleotide concentrations through hydrolysis of cAMP and/or cGMP to their respective nucleoside 5′ monophosphates. The *PDE1A* gene encodes Ca^2+^/calmodulin-dependent PDEs that are activated by calmodulin in the presence of calcium, leading to increased hydrolysis of both cAMP and cGMP [14]. The rs182089527 mutation identified in the current investigation only influenced the function and expression of *PDE1A* isoform 5 (*PDE1A9*), which is manly expressed in the brain tissue [15]. Although we did not conduct further functional analysis on *PDE1A9*, its expression level has been reported to be lower than that of other isoforms [13].

We propose three possible mechanisms contributing to the apparent association of *PDE1A9* with NL. First, *PDE1A9* is the main digestive enzyme for cGMP, and thus deprivation of *PDE1A9* leads to an increase of cGMP levels, which can influence the secretion of H_2_O, Na^−^, Cl^−^, and HCO3^−^ through the renin secretion pathway. More importantly, the change of the acidic environment in tubular epithelial cells may influence the formation of stones in the kidney. This hypothesis is somewhat supported by the present genotype–phenotype correlation analysis, which showed that the levels of creatinine and urea were increased in the urine of patients suffering from NL compared with those of control samples. Second, in addition to PDEase_I_N and the HDc motif, *PDE1A9* also has Zn^2+^- and Mg^2+^-binding sites, and thus depletion of *PDE1A9* can lead to enrichment of Zn^2+^ and Mg^2+^ in the cytoplasm, which could in turn contribute to NL development by damaging cell function or influencing the balance of ions. Third, the complete lack of *PDE1A9* expression in tubular epithelial cells may lead to cystogenesis, resulting in the dysfunction of tubular cells and contributing to the progress of NL. This assumption is based on the following observations: (1) knockout of *PDE1A* changes cAMP signaling in renal development, thereby progressing cystogenesis [16]; (2) in the present study, patients with the rs182089527 mutation showed cystic changes in the kidney and liver, as well as hypertension, typical clinical characteristics of polycystic kidney disease.

In the current study, the MAF of rs182089527 was 0.43%, which was higher than the frequency of 0.26% in data from the 1000 Genomes database, and was also more frequently detected in the NL patients (1.61%). Moreover, the MAF of rs182089527 was found to be close to zero in Europeans and African Americans [17], which may explain the difference in the prevalence of NL between different ethnic groups. However, further larger cohort data are needed to clarify this findings.

The monogenic nature of NL suggested by the present results has practical implications that could help to facilitate personalized treatment. However, there are several questions that remain based on the current investigation. First, the potential mechanism by which the rs182089527 mutation leads to disease requires further research. In particular, the distribution and function of *PDE1A* isoform 5 is unclear, which will be important information to help explain the progress of NL. Second, the relationships among the rs182089527 mutation, NL, and cyst development need to be clarified. Previous case reports have pointed to the coexistence of NL and renal cysts [18]. It is possible that thers182089527 mutation leads to a cystic change, and then causes the development of NL. Although the potential mechanism underlying the association between NL and the rs182089527 mutation requires further investigation in prospective trials, our results highlight the complex and heterogeneous nature of NL, which may facilitate personalized treatment as well as prenatal screening.

## Conclusion

Our study provided genetic evidence that the rs182089527 mutation in *PDE1A* may be involved in the development of NL. Our clinical and molecular findings may contribute to gaining a better understanding of the mechanism underlying NL as well as the relationship between NL and cysts.

## Additional files

Additional file 1: Figure S1-S5. Auxiliary clinical examination and experiment data. (DOCX 624 kb)

Additional file 2: Table S1-S2. Sequence quality statistical and candidate gene list. (DOCX 18 kb)

## Abbreviations

NL: Nephrolithiasis
PDE1A: Phosphodiesterase 1A
SNP: Single nucleotide polymorphism
