# Supplementary material for: PDE1A polymorphism contributes to the susceptibility of nephrolithiasis
Source: BMC Genomics. 2017 Dec 20;18:982. doi: 10.1186/s12864-017-4247-8 (PMC5738135; doi:10.1186/s12864-017-4247-8)

**Additional file Methods**

**Additional file Figure S1**. | Computed tomography (CT) scan of liver shows many cystic dark areas (A). Ultrasonography and CT image of kidney demonstrated cystic dark areas (B), hydronephrosis, and multiple stones in the ureter (C), renal pelvis (D) appear as dense, white objects. Cystic and stones are marked separately by white arrow and red circle.


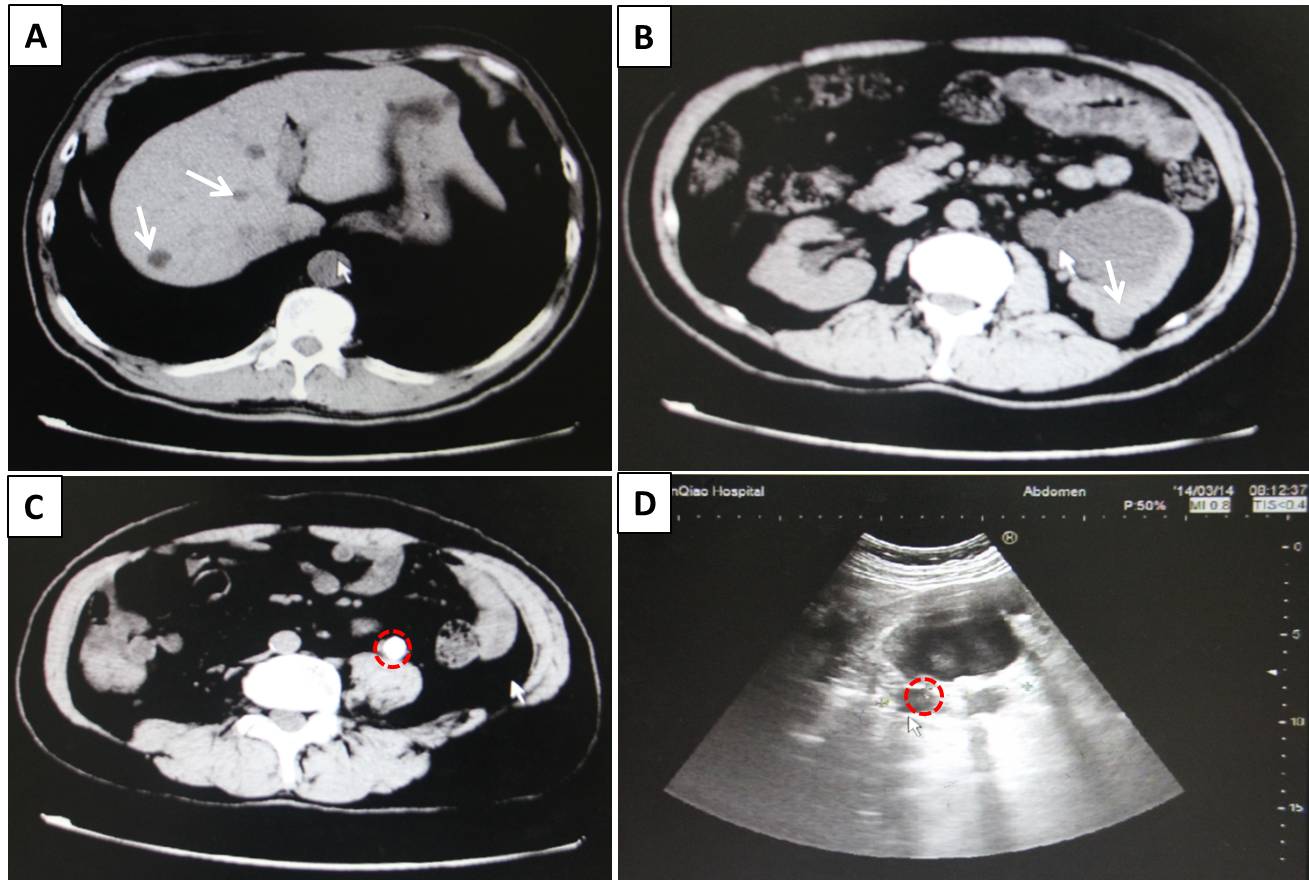


**Additional file Figure S2 |** DNA quality for all 10 sequencing samples conducted by Agarose gel electrophoresis and λDNA-Hind Ⅲ digest.


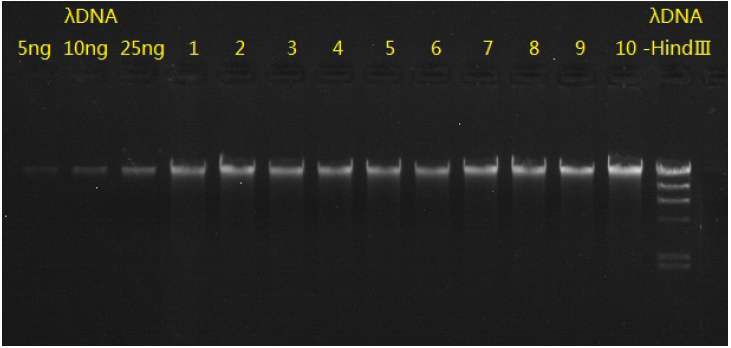


**Additional file Figure S3 |** Identification candidate SNPs in three subjects of affected family. IGV browser view of rs182089527. Top panel shows chromosome with the mutation location. Middle panels depict representative individual reads as well as the relative coverage per base pair for II3, II4 and III3. Sanger sequencing traces for related samples.


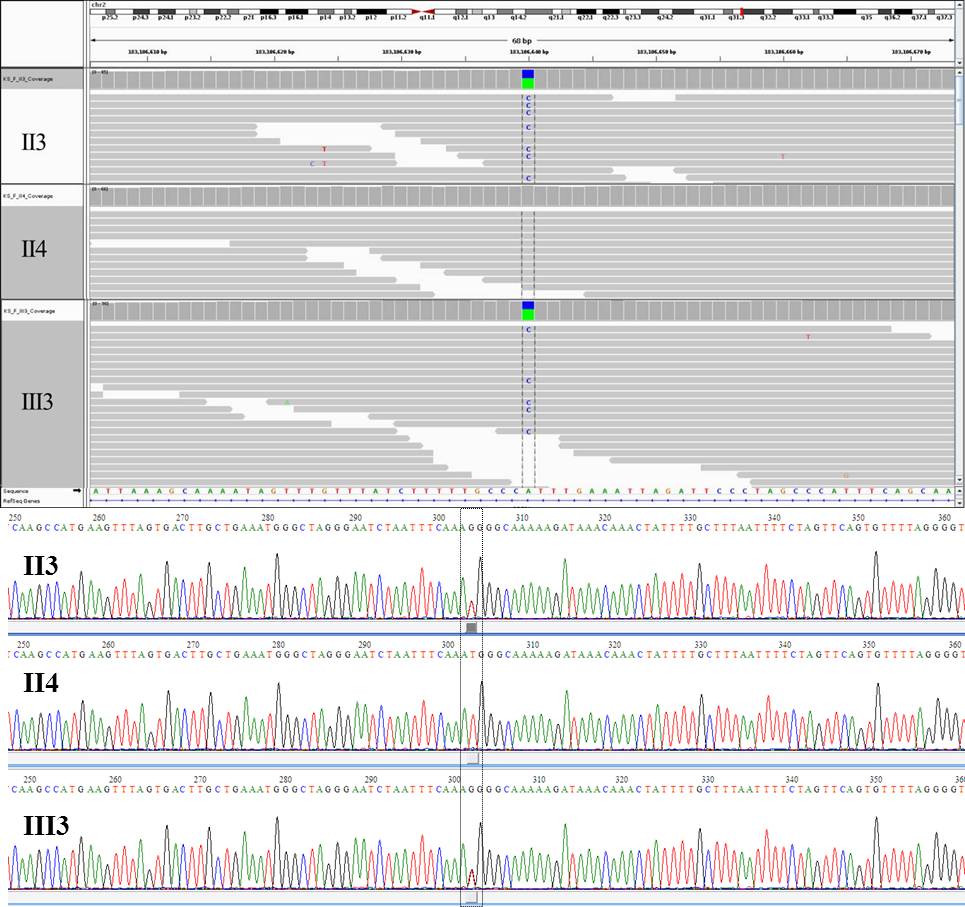


**Additional file Figure S4** | The level of mRNA and protein expression indicated difference between WT (PDE1A^A/A^), mutation type (PDE1A^C/C^) and normal controls (3 samples and 3 biological replications were done, *** P < 0.001).


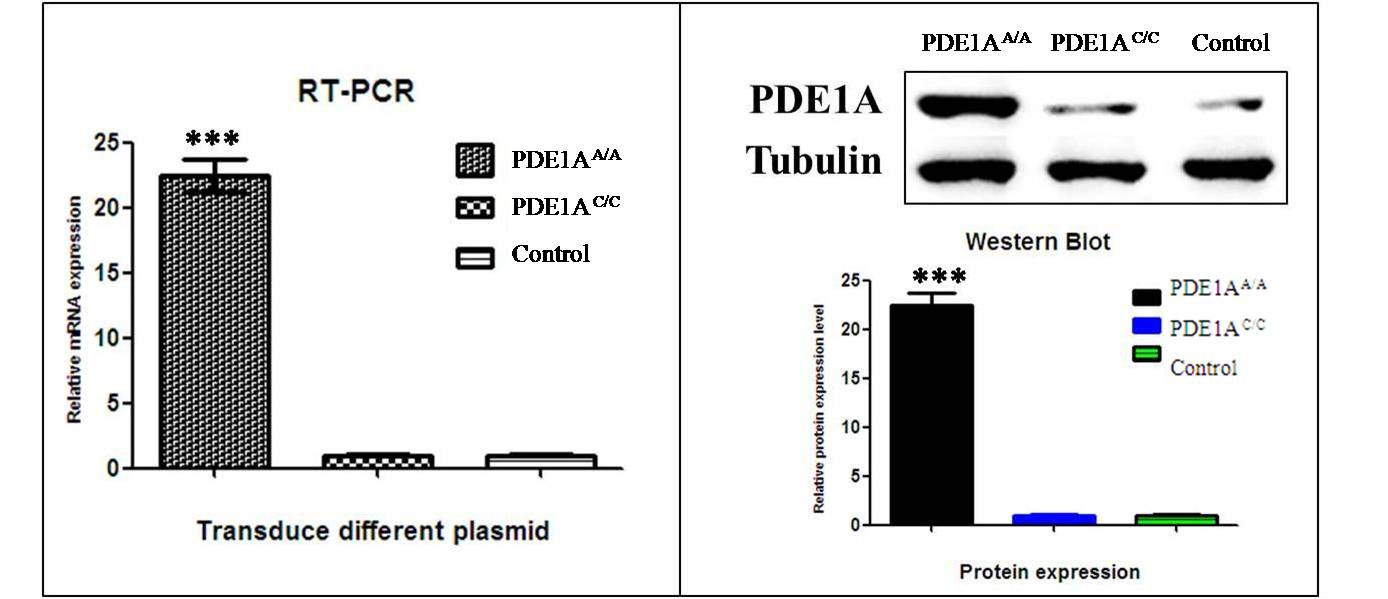


**Additional file Figure S5** | Randomly choose 19 values from 894 values of clinical experimental test in normal controls got an empirical distribution of average values for different clinical experiment lab test. The red line denoted the average values from 19 samples with rs182089527 mutation.


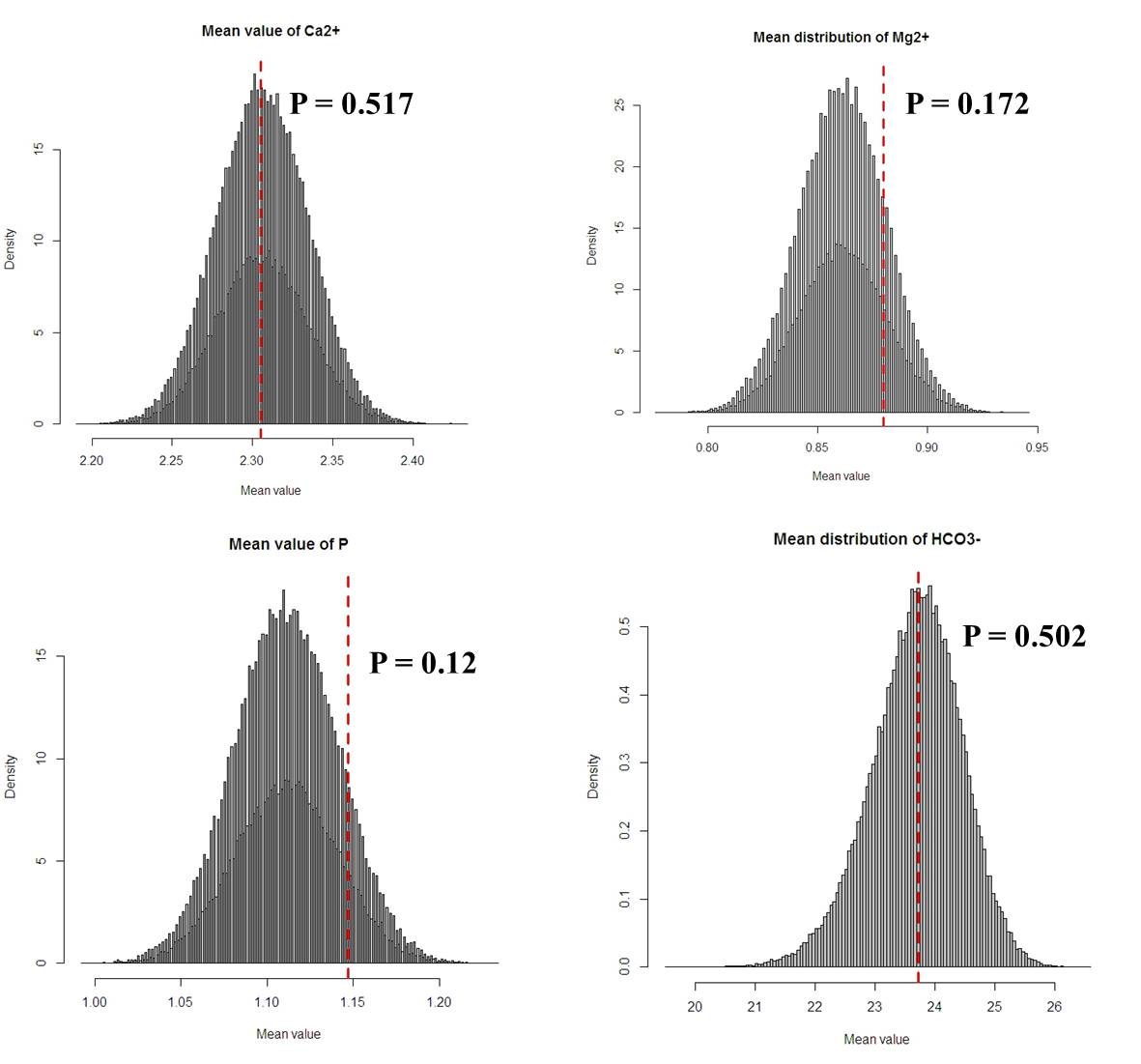

Supplement: Supplementary file 1 — Auxiliary clinical examination and experiment data. (DOCX 624 kb) [file 12864_2017_4247_MOESM1_ESM.docx]
