# Supplementary material for: PDE1A polymorphism contributes to the susceptibility of nephrolithiasis
Source: BMC Genomics. 2017 Dec 20;18:982. doi: 10.1186/s12864-017-4247-8 (PMC5738135; doi:10.1186/s12864-017-4247-8)
Supplement: Supplementary file 2 — Sequence quality statistical and candidate gene list. (DOCX 18 kb) [file 12864_2017_4247_MOESM2_ESM.docx]

| **Additional file Table 1 Exome sequencing data production** | | | | | | | | | | | | |
| --- | --- | --- | --- | --- | --- | --- | --- | --- | --- | --- | --- | --- |
|  | **II3** | **II4** | **III5** | **II6** | **II1** | **II8** | **II7** | **II5** | **III3** | **III4** | **Mean** |  |
| Total_sequenced_base | 5911233236 | 5763632063 | 5149525531 | 5957158100 | 6433526497 | 6574237214 | 6434517245 | 6133779573 | 6598165041 | 6144029772 | 6109980427 |  |
| Total_reads | 60291731 | 58944089 | 52383512 | 60328517 | 64747181 | 66139483 | 64175408 | 62024089 | 68432070 | 64855790 | 62232187 |  |
| Unique_reads_aligned | 58641320 | 57331549 | 50949975 | 58674852 | 63023696 | 64347961 | 62443756 | 60365427 | 66510672 | 63006127 | 60529533.5 |  |
| Per_target>2X | 98.52% | 98.42% | 98.49% | 98.42% | 98.56% | 98.55% | 98.43% | 98.51% | 98.54% | 98.52% | 98.50% |  |
| Per_target>10X | 97.42% | 97.20% | 96.97% | 97.36% | 97.59% | 97.64% | 97.46% | 97.32% | 97.52% | 97.31% | 97.38% |  |
| Per_target>20X | 94.30% | 93.98% | 92.50% | 94.49% | 95.05% | 95.19% | 95.01% | 94.21% | 94.88% | 94.10% | 94.37% |  |
| Per_target>30X | 88.50% | 87.97% | 84.56% | 89.03% | 90.33% | 90.61% | 90.42% | 88.67% | 90.11% | 88.45% | 88.86% |  |
| Per_target>40X | 80.19% | 79.32% | 73.95% | 80.92% | 83.35% | 83.85% | 83.47% | 80.85% | 83.26% | 80.59% | 80.98% |  |
| Per_target>50X | 70.25% | 69.11% | 62.32% | 71.08% | 74.66% | 75.43% | 74.72% | 71.54% | 74.89% | 71.32% | 71.53% |  |
| Per_target>100X | 27.77% | 26.34% | 20.83% | 28.37% | 32.61% | 34.19% | 32.50% | 30.07% | 34.38% | 30.15% | 29.72% |  |
| Mean_depth | 79.27 | 77.29 | 69.06 | 79.89 | 86.28 | 88.16 | 86.29 | 82.26 | 88.48 | 82.39 | 81.937 |  |
| Q20(%) | 98.33% | 98.30% | 98.33% | 98.31% | 98.30% | 98.37% | 98.32% | 98.35% | 98.31% | 99.06% | 98.40% |  |
| Q30(%) | 95.06% | 95.00% | 90.48% | 90.27% | 90.25% | 90.53% | 90.35% | 90.43% | 90.31% | 90.31% | 91.30% |  |
| Ti/Tv_ratio_all | 2.41 | 2.42 | 2.42 | 2.41 | 2.39 | 2.43 | 2.41 | 2.41 | 2.42 | 2.42 | 2.414 |  |
| Ti/Tv_ratio_known | 2.43 | 2.44 | 2.44 | 2.43 | 2.41 | 2.44 | 2.43 | 2.43 | 2.43 | 2.43 | 2.431 |  |
| Ti/Tv_ratio_novel | 1.86 | 1.73 | 1.81 | 1.8 | 1.69 | 1.94 | 1.8 | 1.84 | 1.87 | 1.93 | 1.827 |  |
| Q20 correspondence calling correct probabilities of 99%, Q30 correspondence calling correct probabilities of 99.9%.  Ti/Tv ratio calculation based on GATK software. | | | | | | | | | | | | |
|  |  |  |  |  |  |  |  |  |  |  |  |  |

**Additional file Table 2 Six SNPs within six genes were selected for further validation**

| **Chr** | **Position** | **Allele** | **rsID** | **Gene** | **Function** | **SIFT** | **Polyphen2** | **IsRare** | **Gene_Name** |
| --- | --- | --- | --- | --- | --- | --- | --- | --- | --- |
| 2 | 183106640 | A/C | rs182089527 | PDE1A | startloss | 0 | 0.115 | Y | Phosphodiesterase 1A |
| 2 | 182430816 | G/A | . | CERKL | missense | 0.005 | 1 | Y | Ceramide kinase like |
| 4 | 6616029 | C/T | rs201851950 | MAN2B2 | missense | 0.003 | 0.996 | Y | Mannosidase alpha class 2B member 2 |
| 15 | 75044506 | A/G | . | CYP1A2 | missense | 0.001 | 0.73 | Y | Cytochrome P450 family 1 subfamily A member 2 |
| 17 | 40695867 | C/T | rs199729746 | NAGLU | missense | 0.051 | 0.998 | Y | N-acetyl-alpha-glucosaminidase |
| 18 | 77211776 | C/A | . | NFATC1 | missense | 0.019 | 1 | Y | Nuclear factor of activated T-cells 1 |

Chr: chromosome; SNP function were annotated with Annotation Tool - SNPnexus (<http://www.snp-nexus.org/>); SIFT and Polyphen2 were employed to predict SNP damaging score; All of the SNPs were rare (MAF < 0.1%)
